# Alzheimer’s Biomarkers in the Community: Learnings and Best Practices from the SiteRx Neurology Network

**DOI:** 10.1002/alz.094855

**Published:** 2025-01-09

**Authors:** Daniel Gautieri, Michael Stalder

**Affiliations:** ^1^ SiteRx, New York, CA USA; ^2^ SiteRx, New York, NY USA

## Abstract

**Background:**

Alzheimer’s Disease community care is rapidly evolving as investigative therapies receive approvals and many more investigative molecules continue to show promising late phase data. While interventional approaches have been shown to have real clinical application, implementation of new diagnostics such as blood‐based biomarkers for beta‐amyloid confirmation has lagged in the community setting. This narrative explores Alzheimer’s biomarkers within the SiteRx Alzheimer’s Real‐World Data network, the challenges stakeholders (physicians, patients, and biomarker infrastructure) are facing, and best practices for adoption of continually emerging interventions and diagnostics.

**Methods:**

SiteRx analyzes data related to the uptake of biomarkers (e.g., CSF, PET Amyloid, blood‐based) within SiteRx’s Alzheimer’s Real‐World Data network, along with HCP and practice‐level qualitative insights to identify bottlenecks in initial and sustained adoption of biomarkers in the community‐setting. This mix of primary and secondary data highlights disparities across the community ranging from provider readiness (e.g., awareness or buy‐in) to implementation readiness (e.g., connectedness with diagnostics infrastructure).

**Result:**

While uptake of biomarker use is fragmented in the community‐setting, there is increasing utilization of diagnostics and a growing cohort of biomarker‐positive patients within the SiteRx Alzheimer’s Real‐World Data network. The primary lagging indicator of adoption is Leqembi uptake, with physicians prescribing the mAb administering 5‐10x more diagnostics than. non‐prescribers. Anecdotally, the challenge for low‐to‐no adoption physicians is primarily driven by a lack of clear direction on how to navigate the diagnostics infrastructure. On the other end, high prescribers face mounting difficulty coordinating continued diagnostic requirements for patients on Leqembi due to required support staff, limited availability of PET amyloid appointments, and other factors.

**Conclusions:**

The uptake of Alzheimer’s diagnostics has been slow overall, but continues to increase, especially in certain geographies and practices. As more diagnostic methods emerge, providers are becoming more well‐equipped to understand the disease and develop enhanced care plans for those living with Alzheimer’s disease. In turn, SiteRx’s Alzheimer’s Real‐World Data network is enriched with biomarker‐positive patients, growing the potential pool of interested and suitable patients for breakthrough therapies, promising investigational therapies, and further utilization of disease‐characterizing diagnostics.

